# Layer-by-Layer Assembled Bacterial Cellulose/Graphene Oxide Hydrogels with Extremely Enhanced Mechanical Properties

**DOI:** 10.1007/s40820-018-0195-3

**Published:** 2018-03-27

**Authors:** Honglin Luo, Jiaojiao Dong, Fanglian Yao, Zhiwei Yang, Wei Li, Jie Wang, Xinhua Xu, Jian Hu, Yizao Wan

**Affiliations:** 1 School of Materials Science and Engineering, East China Jiaotong University, Nanchang, 330013 People’s Republic of China; 2 School of Materials Science and Engineering, Tianjin Key Laboratory of Composite and Functional Materials, Key Laboratory of Advanced Ceramics and Machining Technology, Ministry of Education, Tianjin University, Tianjin, 300072 People’s Republic of China; 3 School of Chemical Engineering, Tianjin University, Tianjin, 300072 People’s Republic of China

**Keywords:** Bacterial cellulose, Nanocomposite, Graphene oxide, Biosynthesis, Nanofiber, Hydrogels

## Abstract

Uniform dispersion of two-dimensional (2D) graphene materials in polymer matrices remains challenging. In this work, a novel layer-by-layer assembly strategy was developed to prepare a sophisticated nanostructure with highly dispersed 2D graphene oxide in a three-dimensional matrix consisting of one-dimensional bacterial cellulose (BC) nanofibers. This method is a breakthrough, with respect to the conventional static culture method for BC that involves multiple in situ layer-by-layer assembly steps at the interface between previously grown BC and the culture medium. In the as-prepared BC/GO nanocomposites, the GO nanosheets are mechanically bundled and chemically bonded with BC nanofibers via hydrogen bonding, forming an intriguing nanostructure. The sophisticated nanostructure of the BC/GO leads to greatly enhanced mechanical properties compared to those of bare BC. This strategy is versatile, facile, scalable, and can be promising for the development of high-performance BC-based nanocomposite hydrogels.
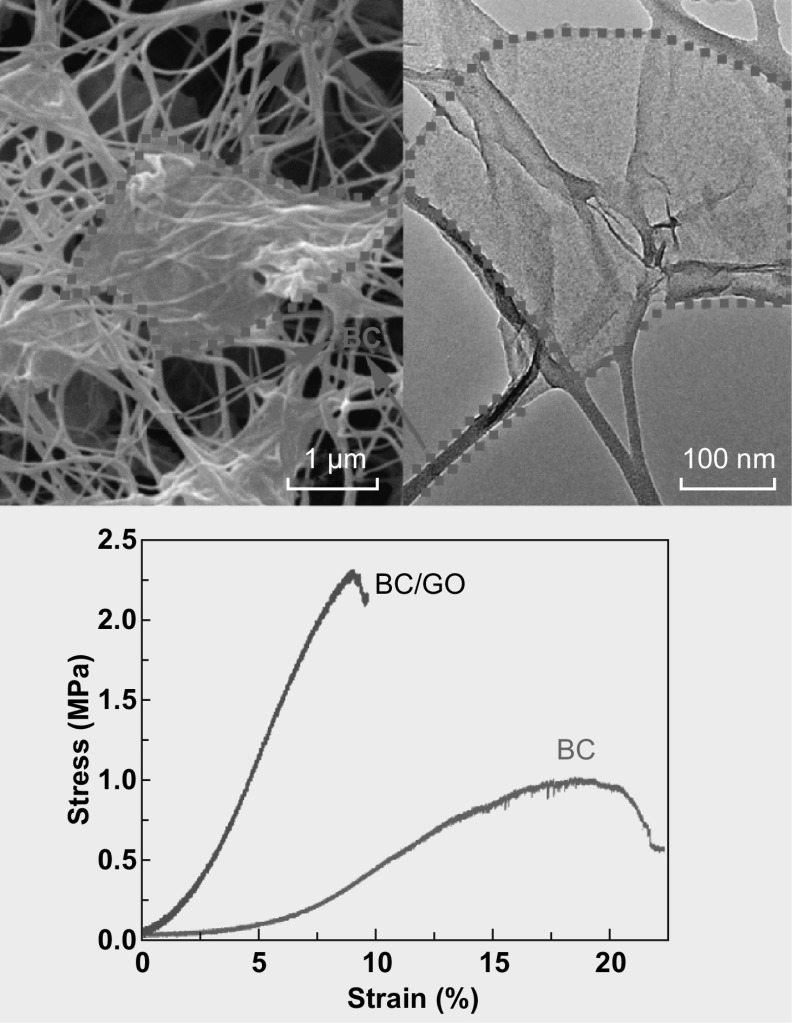

## Highlights

- A modified in situ static culture method (layer-by-layer assembly, LBLA) was developed.
- The LBLA method ensures uniform distribution of graphene oxide (GO) in bacterial cellulose (BC) and makes very thick BC/GO hydrogels with homogeneous structures.
- The BC/GO hydrogels show greatly enhanced mechanical properties over bare BC.

## Introduction

Nano-carbon materials, such as one-dimensional (1D) carbon nanotube (CNT) and two-dimensional (2D) graphene (GE) and graphene oxide (GO), are believed to be promising candidate materials for tissue engineering and regenerative medicine applications owing to their large specific surface area, high porosity, and excellent mechanical properties [1–7]. Among these carbonaceous nanomaterials, GO is considered a promising material for biological applications owing to its excellent biocompatibility, better dispersibility in water than GE, and abundant surface functional groups [8–12]. Furthermore, GO can support and accelerate adhesion, proliferation, and differentiation of various mammalian cells [12–15].

Despite its solubility in water, GO tends to aggregate in physiological environments because of nonspecific binding to proteins [16], which will inevitably lower its reinforcement effectiveness when used as nanofillers in GO-based nanocomposites. To make full use of its good dispersion in water, GO has been used to reinforce a natural polymer, bacterial cellulose (BC), to form BC/GO nanocomposites. For instance, Feng et al. [17] prepared the BC/GO nanocomposite by mechanically mixing BC fragments with a GO aqueous dispersion. The obtained nanocomposites show a tensile strength of 242 MPa in the dry state (5 wt% GO), which is an improvement of 22% compared to that of bare BC (198 MPa). Liu et al. [18] prepared a BC/GO nanocomposite (in the dry state) with a tensile strength of 18.48 MPa by a one-step cross-linking method. In both cases, however, the obtained nanocomposites broke the intrinsically three-dimensional (3D) structure of the BC, which is the most precious feature distinguishing it from other natural polymers. Incorporation of GE or CNTs into the inner core of a 3D BC network, by filtration or post-immersion in a solution of GE or CNTs, is challenging because of the lack of large pores (> 20 μm) in pristine BC [19].

In order to retain the advantageous 3D structure of BC, Yoon et al. [20] reported a post-processing immersion method to fabricate BC-CNT nanocomposites by immersing BC pellicles in CNT solutions. However, this method cannot work when the BC pellicles are thick. More importantly, in the case of GO, the post-processing immersion method might not be feasible for the preparation of BC/GO nanocomposites, since GO is much larger than CNT and thus cannot enter the inner structure of BC pellicles. In our previous studies, a one-pot in situ biosynthesis approach was developed, and a BC/GO nanocomposite with homogeneous GO nanosheets in a BC matrix was successfully fabricated [21]. The BC/GO nanocomposite (in a dry state) showed high mechanical properties and improved electrical conductivity, compared to those of the pristine BC. Unfortunately, neither post-processing immersion nor in situ biosynthesis could enable GO to penetrate the internal area of the BC network when the BC/GO hydrogel was thicker than 2 mm. Therefore, a great deal of effort is required to improve the uniform dispersion of GO in a 3D integrated BC matrix, particularly when thick BC/GO products are required.

Herein, we report a novel in situ layer-by-layer assembly (LBLA) method for fabricating thick (≥ 5 mm) BC/GO nanocomposite hydrogels with highly dispersed GO nanosheets bundled by 3D interconnected BC nanofibers. The sophisticated porous structures and improved mechanical properties of the as-prepared BC/GO nanocomposites were investigated carefully.

## Experimental

### Materials and Methods

Yeast extract, tryptone, disodium phosphate (Na_2_HPO_4_), and acetic acid were used as-received for BC production. A commercially available aqueous dispersion of GO with a concentration of 0.5 mg mL^−1^ was purchased from Nanjing XFNANO Materials Technology Co. Ltd., China. The bacterial strain, *Komagataeibacter xylinus* X-2, was kindly provided by Tianjin University of Science and Technology, Tianjin, China.

### Preparation of BC, c-BC/GO, and BC/GO

Prior to inoculation, the culture medium (pH = 4.5) of BC, composed of 2.5% (w/v) glucose, 0.75% (w/v) yeast extract, 1% (w/v) tryptone, and 1% (w/v) Na_2_HPO_4_, was sterilized at 121 °C for 30 min. This recipe has been reported in our previous work [22, 23]. To prepare BC/GO nanocomposite hydrogels, the GO suspension was added to the above-mentioned culture medium of BC under intense stirring for 60 min. A pure BC membrane (≈ 3 mm in thickness and denoted as BC_0_ hereinafter), prepared by a conventional static culture method, was placed in a culture dish. Afterward, the GO-dispersed culture medium was sprayed onto the surface of the as-obtained BC_0_ membrane, on which new BC grew at the interface of the BC_0_ membrane and the GO-dispersed culture medium, leading to a BC/GO film. When the medium was consumed, additional GO-dispersed culture medium was sprayed onto the surface of the newly prepared BC/GO film, and the second layer of the BC/GO film was produced. The process continued until the desired hydrogel thickness was reached.

The GO content in BC/GO nanocomposites (with respect to hydrated BC) was adjusted by the volume ratio of BC culture medium to GO dispersion (Table 1). Three BC/GO nanocomposite hydrogels, namely BC/GO-1, BC/GO-2, and BC/GO-3, were prepared, which contained 0.11, 0.16, and 0.22% of GO by mass (wt%), respectively (Table 1). The GO contents were obtained by measuring the weight differences of BC and BC/GO hydrogels with identical volumes, since GO addition does not affect the production of BC. For comparison, a BC/GO hydrogel with a thickness of 5 mm was prepared by conventional in situ biosynthesis [21] at a volume ratio of *V*_culture medium_/*V*_GO dispersion_ = 3:1, which was designated as c-BC/GO-2. Additionally, pure BC hydrogels were prepared following the same procedure using a GO-free culture medium. The harvested hydrogels were purified using the previously reported procedure [21], providing BC/GO and BC hydrogels for mechanical testing. For the other characterization methods, the hydrogels were freeze-dried to produce aerogels.

**Table 1:** GO content in various BC/GO nanocomposite hydrogels prepared in this work

Sample No.	*V*_culture medium_/*V*_GO dispersion_	GO content with respect to BC (wt%)
BC/GO-1	5:1	0.11
BC/GO-2	3:1	0.16
BC/GO-3	1:1	0.22
c-BC/GO-2	3:1	–

### Characterization Methods

The photographs were taken with a digital camera. The morphologies of the BC and BC/GO aerogels were observed using a field emission scanning electron microscope (FE-SEM, Nano 430, FEI, USA) and transmission electron microscope (TEM, JEOL, Japan) operating at 200 kV. Fourier transform infrared spectroscopy (FTIR) was performed using a Bruker Optik GmbH spectrometer. X-ray photoelectron spectroscopy was performed with a PHI Quantera X-ray photoelectron spectroscope (XPS, ULVAC-PHI, Inc., Japan). The crystallographic structure was analyzed on a D8 Advance X-ray diffractometer (XRD) using Cu Kα radiation (λ = 0.154 nm) at the scanning range of 10°–50° at a scan speed of 0.02° s^−1^; the data were obtained using the MDI/JADE6 software package attached to the Rigaku XRD instrument. The crystallinity index (CI) was calculated by Segal’s method using Eq. 1 [24]:

1 $${\text{CI}} = (I_{200} - I_{am} )/I_{200}$$

where *I*_200_ is the intensity at a 2*θ* value of 22° and *I*_*am*_ is the intensity of the baseline at a 2*θ* value of 18°.

Thermogravimetric analysis (TGA) was conducted using a TGA instrument (STA449F3) with a heating rate of 10 °C min^−1^ from room temperature to 800 °C. The Brunauer–Emmett–Teller (BET) surface area, pore size, and volume were measured by the nitrogen adsorption method using a surface area analyzer (NOVA 2200e).

Static tensile tests of BC and BC/GO hydrogels (dimensions: 50 × 10 × 2 mm^3^) were conducted using a micro-electromagnetic fatigue testing machine (MUF-1050, Tianjin Care Measure & Control Co., Ltd., Tianjin, China) at a strain rate of 0.1 mm s^−1^. At least five specimens were tested for each sample, and the averages and standard deviations were reported.

## Results and Discussion

**Fig. 1:**
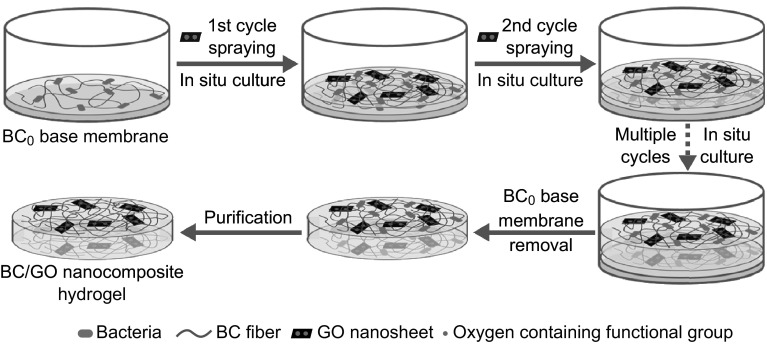
Schematic illustration of the typical fabrication process of BC/GO nanocomposite hydrogel. The mixture of GO suspension and culture medium of BC was sprayed onto the BC_0_ base film (in the first cycle) or the newly grown BC/GO (in the subsequent cycles) followed by in situ culture

The typical fabrication process for the BC/GO nanocomposite hydrogel is schematically illustrated in Fig. 1. Prior to LBLA, a base membrane (a BC_0_ film) was prepared by a conventional static culture method. This BC_0_ base membrane was placed in a container (e.g., a disk). The LBLA method includes numerous cycles, each of which involves two consecutive steps. The first step is to spray the GO-containing culture medium (aqueous suspension, ≈ 0.5 mL) as droplets onto the surface of the BC_0_ base membrane such that the LBLA process (namely biosynthesis, the second step) initiates, resulting in the growth of a BC/GO film (≈ 0.2–0.4 mm in thickness) on the surface of the base membrane with the help of sterilized oxygen. In the second cycle, the resultant BC/GO film serves as a new base membrane on which the second BC/GO film (the same thickness as the first BC/GO film) grows via biosynthesis. These cycles repeat until a predefined hydrogel thickness (≈ 2 mm for tensile testing and ≈ 5 mm for morphology analysis) is reached. The last step is the removal of the base membrane and purification of the hydrogel, leaving a freestanding BC/GO nanocomposite hydrogel.

**Fig. 2:**
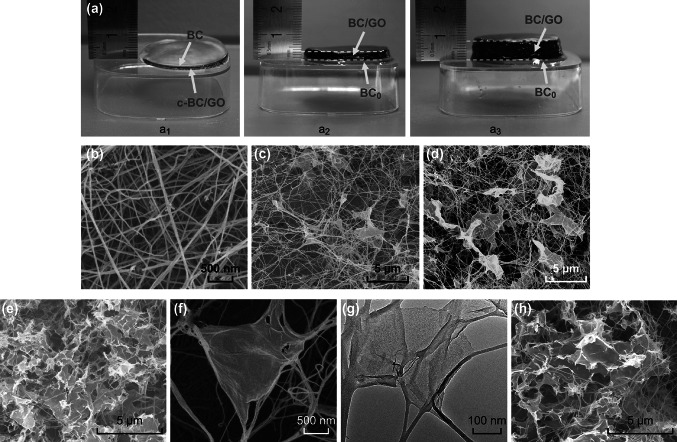
**a** Digital photographs of c-BC/GO and BC/GO hydrogels. SEM images taken from the surface layers of **b** BC, **c** BC/GO-1, **d** BC/GO-2, and **e** BC/GO-3. **f** Magnified SEM and **g** TEM images of the “leaf and vein” nanostructure of BC/GO-2. **h** A representative SEM image of BC/GO-3 from central area indicating the uniform structure

Figure 2 shows the photographs of the c-BC/GO-2 and BC/GO-2 hydrogels and the morphology of the BC and BC/GO aerogels. As shown in Fig. 2a, the digital photograph of c-BC/GO clearly demonstrates that there is a GO-free thin layer on top of the c-BC/GO sample (Fig. 2a_1_), which suggests that the maximum thickness of a c-BC/GO composite is ≈ 2 mm for conventional in situ biosynthesis. This is likely due to the gradual decrease in the GO content in the culture medium during the fermentation process; however, the exact mechanism is not yet fully understood. In contrast, a uniform black BC/GO-2 hydrogel is noted with a thickness of either 3 mm (Fig. 2a_2_) or even 7 mm (Fig. 2a_3_), which indicates that the LBLA strategy can produce BC/GO hydrogels with uniformly distributed GO. To further observe the distribution of GO in the BC matrix, SEM and TEM analyses were conducted. Figure 2b displays a typical 3D porous interconnected nanostructure of BC, which is similar to that reported in our previous work [21, 25], indicating that the LBLA process does not affect the morphology of the resultant BC. As seen in Fig. 2c–e, GO is uniformly distributed in the BC network in BC/GO-1, BC/GO-2, and BC/GO-3. Figure 2f reveals a typical entangled structure. To further examine the morphology of BC/GO nanocomposites, TEM observation was performed that further confirmed the entangled structure (Fig. 2g). Figure 2h shows a typical image of BC/GO-2 from the central part, which demonstrates the uniform distribution of GO in the core area of BC/GO-2. This finding further shows the great capability of the LBLA method in dispersing GO nanosheets throughout the BC matrix. It should be pointed out that higher than 0.22 wt% GO content will result in GO aggregation in BC/GO aerogels owing to the ultra-thinness of GO nanosheets. Therefore, in this work, 0.22 wt% is the highest GO content observed for the BC/GO nanocomposite hydrogels.

**Fig. 3:**
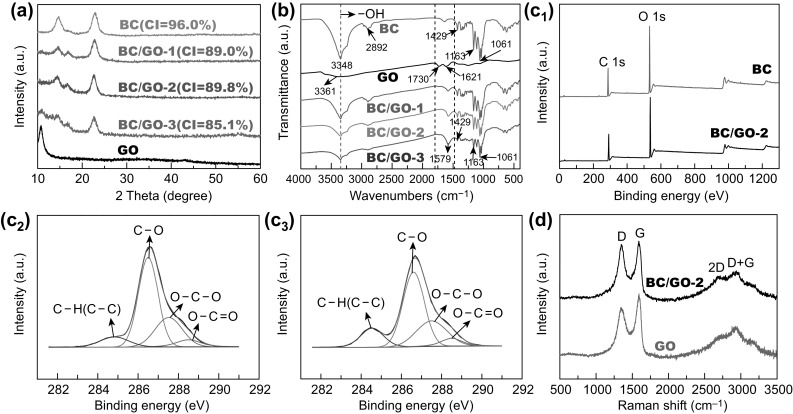
**a** XRD patterns of BC_0_, BC, and BC/GO nanocomposites. **b** FTIR spectra of BC, GO, and BC/GO nanocomposites. **c** Wide-scan XPS spectra of BC and BC/GO-2 (c_1_), high-resolution C 1s spectra of BC (c_2_) and BC/GO-2 (c_3_). **d** Raman spectra of pristine GO and BC/GO-2 nanocomposite

Figure 3a shows the XRD patterns of BC/GO nanocomposites, GO, and BC. It is noted that the positions (14.6°, 16.8°, and 22.8°) of three characteristic peaks, namely the (1$\overline{1}$0), (110), and (200) BC diffraction planes, do not change in the XRD patterns of the BC/GO nanocomposites as compared to BC, indicating that the crystalline structure (lattice parameters) of BC remains unchanged upon incorporation of GO in the culture medium of the BC. However, the crystallinity indices (*CI*) of the three BC/GO samples are significantly lower than that of BC (Fig. 3a). This result indicates that the presence of GO in the BC culture medium has an effect on the formation process of BC crystals, which is generally ascribed to the disturbance by additives to the formation of BC nanofibrils [17, 26, 27]. The smaller *CI* of BC/GO-3 versus those of BC/GO-1 and BC/GO-2 indicates that the GO content has an impact on CI. However, how the concentration of additives affects the formation process is unclear. It is interesting to note that there is no GO peak in the XRD patterns of BC/GO-1 and BC/GO-2, which may indicate even distribution of GO in the 3D BC matrix [25, 28–31], consistent with SEM observation. However, a weak peak is located at ≈ 11° in the XRD pattern of BC/GO-3, which is the same as the intense peak in the XRD pattern of bare GO. The presence of this peak may suggest slight GO aggregation in BC/GO-3, although SEM cannot clearly show this trend.

Surface chemistry of the BC/GO nanocomposites was examined by FTIR analysis (Fig. 3b). In the spectrum of BC, characteristic peaks (3348, 2892, 1429, and 1061 cm^−1^ due to –OH bonds, asymmetric stretching vibration of C–H, asymmetric angular deformation of C–H bonds, and antisymmetric bridge stretching of C–O–C, respectively [18, 32–34]) are observed. In the spectrum of GO, three peaks, centered at 3361, 1730, and 1621 cm^−1^, corresponding to the stretching vibrations of O–H, C=O, and C=C bonds [17, 35], respectively, are observed. The major characteristic absorptions of GO and BC are also noted in the BC/GO nanocomposites. It is noted that the peak intensity of the –OH group (3348 cm^−1^) decreases with increasing GO content in the BC/GO nanocomposites as compared with that observed with pure BC. In addition, an intense peak at 1579 cm^−1^ is noted, and its intensity increases with the GO content. This may indicate a strong interaction (hydrogen bonding) between BC and GO, which causes downshift of the GO C=O group band. The formation of hydrogen bonding is important for enhancing the mechanical properties of BC/GO composites [36, 37].

To further determine the surface chemistry of the BC/GO nanocomposites, XPS analysis was carried out, and the results are presented in Fig. 3c. Both BC and BC/GO-2 show almost identical wide-scan spectra (Fig. 3c_1_). Furthermore, it seems that the C 1*s* spectra of BC and BC/GO-2 are the same, both showing four peaks of C–C or C–H (284.6 eV), C–O (286.5 eV), O–C–O (287.5 eV), and O–C=O (288.5 eV). However, a careful comparison reveals a difference in the sub-peak intensity of C–C or C–H. As expected, the incorporation of GO improves the C–C or C–H peak strength of BC/GO-2.

Figure 3d presents the Raman spectra of GO and BC/GO-2. The two curves are similar, both showing a D band (1344 cm^−1^, which corresponds to the disordered structure of GO sheets) and a G band (1595 cm^−1^, which represents the first-order scattering of the *E*_2g_ vibrational mode) [38, 39]. However, determination of the intensity ratio of D band to G band, *I*_D_/*I*_G_, reveals a significant difference between GO (0.86) and BC/GO-2 (0.97). The improvement in *I*_D_/*I*_G_ of BC/GO-2 over GO can be ascribed to the removal of some oxygen-containing functional groups on the surface of GO [40], since GO can be deoxygenated in alkaline solutions [41] during sample preparation (boiled with a 0.5-M NaOH solution for 15 min and cleaned with 1 wt% NaOH for 2 days), in line with our previous work [21]. As shown in Fig. 3d, besides the D and G bands, two peaks located at 2680 and 2931 cm^−1^ are also noted in the spectra of GO and BC/GO-2, which can be assigned to the 2D and D + G bands, respectively [42]. The 2D band is highly sensitive to the stacking of graphene, and the intensity ratio of 2D band to G band (*I*_2D_/*I*_G_) is an important parameter to evaluate the layering of graphene materials [42, 43]. The *I*_2D_/*I*_G_ ratios of single-, double-, triple-, and multi- (> 4) layer sheets are typically > 1.6, ~ 0.8, ~ 0.30, and ~ 0.07, respectively [43]. The calculated *I*_2D_/*I*_G_ is 0.66 and 0.68 for GO and BC/GO-2, respectively, which suggests a few-layered texture of GO in BC/GO-2, in accordance with the specification of the providers of the GO suspension.

**Fig. 4:**
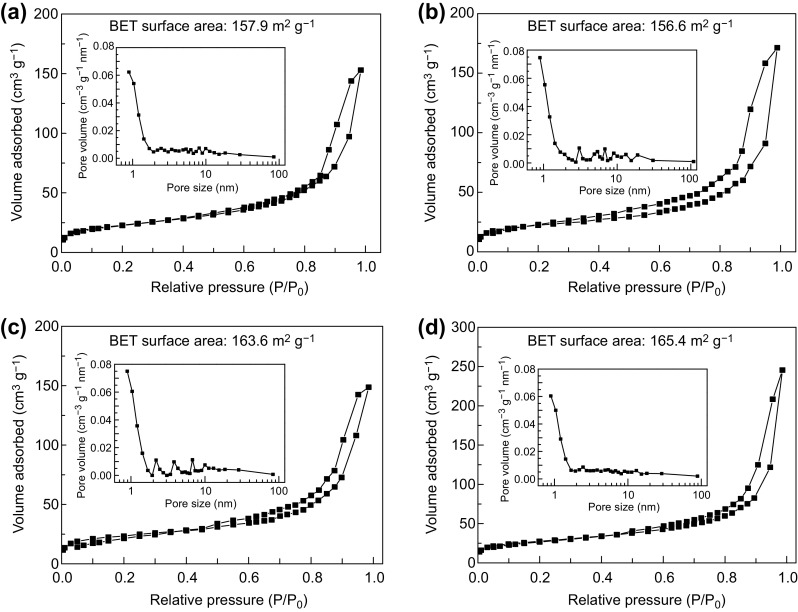
The nitrogen adsorption/desorption curves and pore size distribution curves of **a** BC, **b** BC/GO-1, **c** BC/GO-2, and **d** BC/GO-3

The pore structures of the BC/GO nanocomposites and pure BC (in aerogel) were analyzed by N_2_ adsorption–desorption measurements (Fig. 4). Note that both BC/GO and BC exhibit a characteristic type IV isotherm, suggesting the presence of many mesopores in each material, which can be further evidenced by the pore size distribution shown in the insets of Fig. 4a–d. The BET specific surface areas of BC, BC/GO-1, BC/GO-2, and BC/GO-3 are 156.6, 157.9, 163.6, and 165.4 m^2^ g^−1^, respectively. These results indicate that the incorporation of GO does not significantly affect the BET specific surface area of BC.

**Fig. 5:**
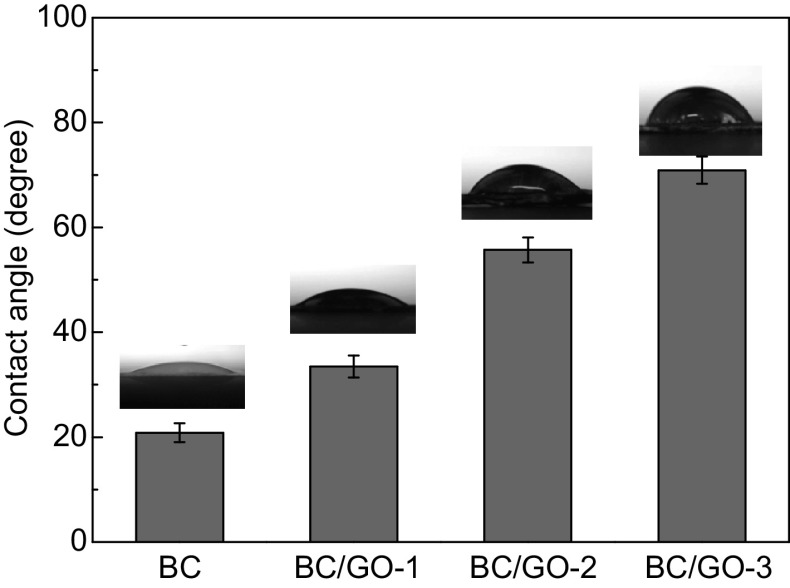
Water contact angles of BC and BC/GO nanocomposites

Surface hydrophilicity of the BC/GO nanocomposites was assessed by measuring their water contact angles (Fig. 5). As expected, the water contact angles of the BC/GO nanocomposites show an increasing trend with the GO content of the BC/GO nanocomposites. This indicates that the incorporation of GO into BC decreases the hydrophilicity of the BC/GO nanocomposites. However, all BC/GO nanocomposites demonstrate favorable hydrophilicity (water contact angles < 90°).

**Fig. 6:**
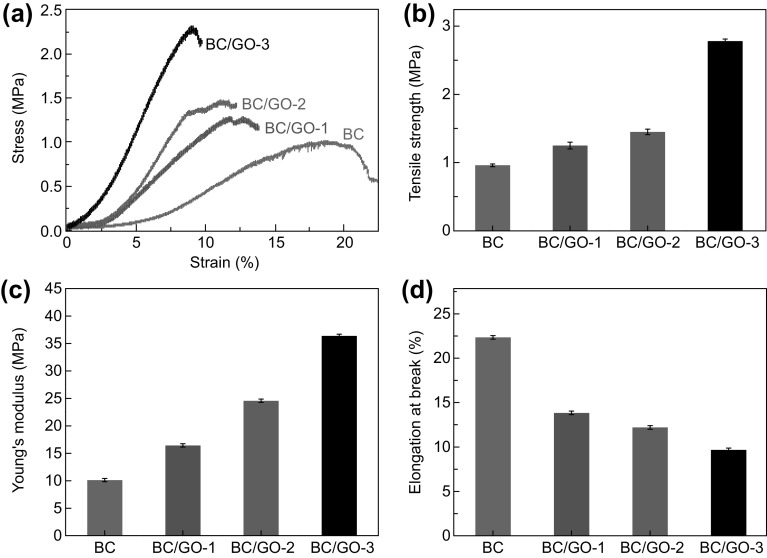
Typical **a** stress–strain, **b** tensile strength, **c** tensile modulus, and **d** strain at break of BC and BC/GO nanocomposites

Mechanical properties of BC and BC/GO hydrogels were tested under tensile loads (Fig. 6). The typical tensile stress–strain curves shown in Fig. 6a reveal an increasing trend in the peak load and decreasing trend in strain at break with the increase in GO content. Data presented in Fig. 6b, c demonstrate that the tensile strength and modulus of BC/GO nanocomposite hydrogels are greatly improved over those of bare BC, and the improvements are dependent on the GO content. Figure 6d shows the opposite trend in strain at break with the GO content as compared to the tensile strength and modulus, as expected. Among the three BC/GO samples, BC/GO-3 has the highest tensile strength (2.78 ± 0.03 MPa) and modulus (36.4 ± 0.3 MPa), which are 2.9 and 3.6 times those of bare BC, respectively. These improvements are much higher than the increases in BC/0.19 wt% GO (3.7% and 32.2%, respectively) and BC/0.29 wt% GO (21.4% and 62.7%, respectively) prepared by conventional static culture [21]. Furthermore, the improvements are also higher than other reported data. For instance, Liang et al. [44] observed a 76% increase in tensile strength and a 62% improvement in Young’s modulus when 0.7 wt% GO was added in a poly(vinyl alcohol) matrix. Zhang et al. [45] claimed a 132% increase in tensile strength and a 36% improvement in compressive strength when 0.8 wt% GO was incorporated in a poly(vinyl alcohol) hydrogel.

**Fig. 7:**
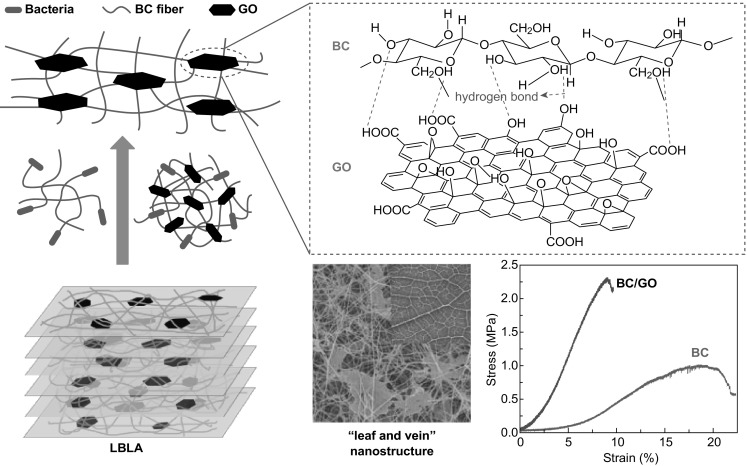
Schematic diagram of “leaf and vein” nanostructure of BC/GO prepared by LBLA strategy. The layer-by-layer culture mode facilitates the bundling of 2D GO nanosheets by 1D BC nanofibers and promotes distribution of 2D GO nanosheets in 3D BC matrix

Such immense improvements in LBLA-derived BC/GO hydrogels can be explained by the following factors (Fig. 7). Firstly, the formation of hydrogen bonding between BC and GO, as revealed by FTIR shown in Fig. 3b, ensures tight bonding between the 1D and 2D components. Secondly, the layer-by-layer culture mode improves the dispersion of 2D GO nanosheets in the 3D BC matrix. Finally, the layer-by-layer culture mode facilitates the mechanical bundling of 2D GO nanosheets by the 1D BC nanofibers, which results in a leaf/vein-like structure. Strong hydrogen bonding, close mechanical bundling, and even distribution are responsible for the tremendous improvements in mechanical properties.

## Conclusions

BC/GO nanocomposites with a sophisticated nanostructure have been fabricated via a novel in situ LBLA strategy. The LBLA method involves two simultaneous steps: self-assembly of 1D BC nanofibers into a 3D structure and self-bundling of 2D GO nanosheets by 1D BC nanofibers. The BC/GO hydrogels show greatly improved mechanical properties over those of bare BC and other BC/GO counterparts prepared by conventional static culture methods. The intriguing nanostructure with strong hydrogen bonding, close mechanical bundling, and even distribution of 2D GO nanosheets throughout the 3D BC network are the main reasons why the LBLA-derived BC/GO hydrogels are ultra-strong. These results show that such a novel LBLA strategy has great promise in the development of high-performance BC-based hydrogels for various applications.
